# In vitro activity effects of combinations of cephalothin, dicloxacillin, imipenem, vancomycin and amikacin against methicillin-resistant *Staphylococcus *spp. strains

**DOI:** 10.1186/1476-0711-5-25

**Published:** 2006-10-12

**Authors:** Guadalupe Miranda-Novales, Blanca E Leaños-Miranda, Mariano Vilchis-Pérez, Fortino Solórzano-Santos

**Affiliations:** 1 Unidad de Investigación en Epidemiología Hospitalaria, Coordinación de Investigación en Salud, Instituto Mexicano del Seguro Social. Mexico City, Mexico; 2 Departamento de Infectología/Dirección Médica, Hospital de Pediatría, Centro Médico Nacional, Siglo XXI, Instituto Mexicano del Seguro Social. Mexico City, Mexico

## Abstract

**Background:**

combinations of drugs has been proposed as an alternative for oxacillin-resistant staphylococci infections, however, limited information about *in vitro *combinations are available for multi-resistant strains. The objective of this study was to describe the interaction of beta-lactams in combination with vancomycin or amikacin against 26 oxacillin and amikacin-resistant nosocomial *Staphylococcus *spp. isolates.

**Methods:**

activity of dicloxacillin plus amikacin, cephalothin plus amikacin, cephalothin plus vancomycin, imipenem plus vancomycin and vancomycin plus amikacin was evaluated by checkerboard synergy tests and the fractional inhibitory concentration index (FIC) was calculated. Results: dicloxacillin plus amikacin, and cephalothin plus amikacin were synergistic or partially synergistic in 84.6% and 100% respectively. For nearly half of the isolates the mean concentrations of dicloxacillin, cephalothin and amikacin at which FIC indexes were calculated were achievable therapeutically. Vancomycin plus amikacin had synergistic effect only against two isolates, and partially synergistic in 38.6%. For the combinations vancomycin plus cephalothin and vancomycin plus imipenem the effect was additive in 76.9% and 80.7% respectively.

**Conclusion:**

in this study the checkerboard analysis showed that amikacin in combination with cephalothin or dicloxacillin was synergistic against most of the resistant strains of *S. aureus *and coagulase-negative *Staphylococcus*. Vancomycin in combination with a beta-lactam (cephalothin or imipenem) showed additivity. An indifferent effect predominated for the combination vancomycin plus amikacin. Even though a synergistic effect is expected when using a beta-lactam plus amikacin combination, it is possible that the effect cannot be clinically achievable. Careful selection of antimicrobial combinations and initial MICs are mandatory for future evaluations.

## Background

Nosocomial staphylococcal infections are a health problem in different countries [1,2]. In the United States of America there has been an increase in methicillin-resistant *Staphylococcus aureus *(MRSA) nosocomial infections, from 2.1% to 35% in a 25-year period [3], with similar information from other developed countries like Japan and Canada [4,5]. In Mexico, antimicrobial resistance reports about the frequency of MRSA and methicillin-resistant (MR) coagulase-negative staphylococci (CoNS) are few, with variable percentages between 20% and 60% [6-10].

Glycopeptides are considered the standard treatment for infections due to MR *Staphylococcus *spp. strains, some authors have expressed their concern about *in vitro *vancomycin MICs and clinical outcomes in patients with MRSA bacteremia [11], thus other available alternatives are being considered (linezolid, tigecycline, daptomycin).

In Mexico, and other Latin American countries, vancomycin was introduced in the early 90's and newer antibiotics are expensive and available only in specialty hospitals. Different antimicrobial combinations are prescribed regularly; some of them include dicloxacillin or nafcillin plus amikacin, cephalothin plus amikacin, and vancomycin plus amikacin; however limited information about "*in vitro*" or *"in vivo" *efficacy for these combinations is available, particularly against nosocomial resistant isolates.

The objective of this study was to describe the interaction of beta-lactams in combination with vancomycin or amikacin against 26 oxacillin and amikacin-resistant nosocomial *Staphylococcus *spp. isolates.

## Methods

### Bacteria

94 *Staphylococcus *isolates obtained from blood and sterile fluids over a 7-month period from 2001 to 2003 were stored at -70°C. The organisms were identified by conventional methods (colonial morphology, Gram stain, and catalase and coagulase tests). Species identification was performed by using the API Staph system (Biomeriéux, L'Etole, France). Antibiotic susceptibility was performed with a broth microdilution method in accordance with the CLSI [12]. The antimicrobials tested included dicloxacillin (UPS-189009), cephalothin (Sigma Chemical Co, St. Louis, Mo. USA), imipenem (Merck, Sharp and Dohme, USA) amikacin (UPS-01950-8) and vancomycin (UPS 70900-7). Oxacillin (UPS-48100-0) susceptibility was performed by Mueller-Hinton broth supplemented with 2% of NaCl. Resistance was corroborated by detecting the *mec A *gene by PCR, by the method previously described [13,14]. Reference type strains included for quality control were: *S. aureus *ATCC # 29213 and *S. aureus *# 43300. For the entire collection oxacillin resistance was detected in 48.5% and 93.1% respectively for *S. aureus *and CoNS.

Twenty six isolates, with resistance to oxacillin and intermediate resistance or resistance to amikacin were selected for the synergy tests. Strain identity was established by pulsed-field gel electrophoresis (PFGE) [15], only single, unrelated strains were included.

### Synergy tests

The checkerboard technique was performed [16,17], including the combinations: dicloxacillin/amikacin, cephalothin/amikacin, cephalothin/vancomycin, imipenem/vancomycin and vancomycin/amikacin. Stock solutions were prepared according to published standards [12].

Synergy tests were performed in 96-well microtiter plate containing two antimicrobial agents in two fold dilutions dispensed in a checkerboard fashion on the day of the assay. Each well contained 0.1 mL of individual antimicrobial combinations. Suspensions with turbidities equivalent to that of a 0.5 McFarland standard were prepared to yield a final inoculums of 3 × 10^5 ^to 5 × 10^5 ^CFU/mL. MICs were read after overnight incubation at 35°C. Growth and sterility controls were included in each plate. Each isolate was tested twice.

Amikacin, dicloxacillin, and cephalothin concentrations tested were from 0.125 to 1026 mg/L, and for vancomycin from 0.06 to 8 mg/L.

### Synergy tests interpretation

For the first clear well in each row of the microtiter plate containing both antimicrobial agents, the fractional inhibitory concentration (FIC) was calculated as follows: FIC of drug A (FIC_A_) = MIC of drug A in combination/MIC of drug A alone, and the FIC of drug B (FIC_B_) = MIC of drug B in combination/MIC of drug B alone. If the MIC of any agent alone occurred at the lowest or highest concentration tested, the FIC was considered not determinable and synergy could not be assessed. The suma of both FICs in each well was used to classify the combination of antimicrobial agents as synergistic effect when FIC indexes were ≤ 0.5; partial synergy FIC >0.5 but < 1; additive FIC = 1.0; indifferent effect when values were >1 and < 4 and antagonistic when values were ≥ 4.0 [18].

## Results and discussion

Resistance *in vitro *was 88.44% to dicloxacillin, 80.7% to cephalothin, 69.23% to imipenem, and 100% to amikacin (11.5% intermediate and 88.4% resistant) for the 26 isolates. All isolates were susceptible to vancomycin.

Results of the checkerboard synergy testing are summarized in table 1. For most of the isolates the combination cephalothin plus amikacin or dicloxacillin plus amikacin showed a FIC < 1. When initial MICs were compared with those registered in the antimicrobial combination, a drop up to 10–12 dilutions in the checkerboard assays was found for beta-lactams. For nearly half of the isolates the mean concentrations of dicloxacillin, cephalothin and amikacin at which FIC indexes were calculated were achievable therapeutically. Combinations that included vancomycin and a beta-lactam had FICs ≥ 1 and 2.

**Table 1:** MICs (mg/L) and FIC indexes of the 26 methicillin-resistant *Staphylococcus *spp.

**SPECIES**	**OX**	**DX**	**CEF**	**IMP**	**VAN**	**AK**	**DX/AK**^**a**^	**FIC**	**CEF/AK**^**b**^	**FIC**	**CEF/VAN**^**c**^	**FIC**	**IMP/VAN**^**d**^	**FIC**	**VAN/AK**^**e**^	**FIC**
*S. aureus*	32	1	64	256	1	32	0.5/4	0.63	0.5/1	0.037	64/1	2	256/1	2	0.5/8	0.75
*S. aureus*	16	128	64	8	1	32	8/4	0.18	8/1	0.16	32/0.5	1	4/0.5	1	0.5/8	0.75
*S. aureus*	>32	32	64	8	1	512	8/128	0.5	8/64	0.25	64/1	2	4/0.5	1	1/64	1.12
*S. aureus*	>32	512	256	1	0.5	64	0.125/32	0.5	2/16	0.26	1/0.5	1	0.5/0.25	1	0.125/32	0.5
*S. aureus*	>32	512	64	512	2	128	2/4	0.03	1/64	0.52	64/2	1	256/1	1	0.125/32	0.31
*S. aureus*	8	32	32	8	2	128	8/32	0.5	4/64	0.62	32/2	2	8/2	2	2/16	1.12
*S. aureus*	8	2	0.5	8	1	128	1/64	1	0.125/64	0.75	0.25/0.5	1	4/0.5	1	1/64	1.5
*S. aureus*	>32	512	2	256	1	512	0.125/4	0.5	0.5/64	0.75	1/0.5	1	128/0.5	1	1/128	2
*S. aureus*	>32	128	128	8	2	128	0.5/32	0.25	16/64	0.52	1/2	1	4/1	1	0.5/64	0.75
*S. aureus*	>32	512	512	1	2	512	0.125/128	0.5	32/256	0.56	128/1	1	0.5/1	1	0.5/128	0.5
*S. epidermidis*	>32	512	4	256	0.5	32	1/8	0.25	0.125/8	0.28	2/0.25	1	128/0.25	1	0.06/0.125	0.12
*S. epidermidis*	>32	512	256	512	2	512	0.125/512	1	8/128	0.28	0.125/2	1	256/1	1	0.125/256	0.56
*S. epidermidis*	>32	512	32	512	1	128	0.125/64	0.5	0.25/64	0.5	0.125/1	1	256/0.5	1	0.125/64	0.62
*S. epidermidis*	>32	512	128	512	1	512	0.5/128	0.25	0.125/256	0.5	0.25/1	1	256/0.5	1	0.125/512	1.12
*S. epidermidis*	>32	512	256	512	1	128	2/64	0.5	1/64	0.5	128/0.5	1	256/0.5	1	1/16	1.12
*S. epidermidis*	>32	512	4	512	1	128	1/64	0.5	0.125/64	0.53	2/0.5	1	256/0.5	1	1/16	1.12
*S. epidermidis*	>32	8	2	512	1	128	4/64	1	0.125/64	0.56	0.125/1	1	256/0.5	1	0.125/64	0.62
*S. epidermidis*	>32	512	4	512	1	64	0.5/16	0.25	0.25/32	0.56	0.25/1	1	256/0.5	1	0.125/64	1.12
*S. haemolyticus*	>32	512	256	512	0.5	64	128/32	0.5	8/64	0.53	256/0.5	2	256/0.25	1	0.5/64	1.5
*S. haemolyticus*	>32	256	512	512	2	128	1/64	0.5	2/64	0.5	256/1	1	256/1	1	2/16	1.12
*S. haemolyticus*	>32	512	512	512	4	256	2/64	0.25	128/128	0.75	256/2	1	512/4	2	4/16	1.12
*S. haemolyticus*	>32	512	512	512	2	256	2/256	1	16/128	0.53	512/2	2	512/2	2	2/128	1.5
*S. hominis*	>32	128	64	256	4	128	2/64	0.5	16/64	0.75	32/2	1	128/2	1	1/64	0.75
*S. hominis*	>32	256	256	256	4	512	2/256	0.5	16/256	0.56	128/2	1	128/2	1	4/256	1.5
*S capitis*	>32	64	2	512	2	128	1/32	0.25	0.25/32	0.37	1/1	1	256/1	1	1/32	0.75
*S. sciuri*	>32	128	128	8	2	64	1/16	0.25	2/32	0.5	128/2	2	8/2	2	2/8	1.12

MIC in combination for a = dicloxacillin plus amikacin, b = cephalothin plus amikacin, c = cephalothin plus vancomycin, d = imipenem plus vancomycin and e = vancomycin plus amikacin.

According to the FIC (table 2), dicloxacillin with amikacin showed synergistic activity against 34.6% and partially synergistic activity in 50% of the isolates, and additive activity against the remainder four (15.6%), cephalothin with amikacin was synergistic against 26.9% and partially synergistic against the rest (73.07%). For cephalothin plus vancomycin combination, the effect was additive against 76.9% (20/26), and indifferent for 23.1%, imipenem plus vancomycin combination showed additivity against 80.7% (21/26) of the isolates and indifference against five isolates. Finally, vancomycin and amikacin combination was synergistic only in two isolates and partially synergistic against 38.46%, and indifferent effect was shown against 53.8% (14/26). None of the combinations showed an antagonistic effect.

**Table 2:** Antimicrobial combinations for reaction for synergy, partial synergy, additivity and indifference against 26 methicillin-resistant *Staphylococcus *spp. isolates.

	**No. of isolates**			
**Antibiotic combinations**	**Synergy**	**Partial Synergy**	**Additivity**	**Indifference**
DK-AK^a^	9	13	4	0
CEF-AK^b^	7	19	0	0
CEF-VAN^c^	0	0	20	6
IMP-VAN^d^	0	0	21	5
VAN-AK^e^	2	10	0	14

^a^DX-AK dicloxacillin plus amikacin.

^b^CEF-AK cephalothin plus amikacin.

^c^CEF-VAN cephalothin plus vancomycin.

^d^IMP-VAN imipenem plus vancomycin.

^e^VAN-AK vancomycin plus amikacin

Multirresistant *Staphylococcus *strains are a common problem [4,10]. Reports of vancomycin tolerant or resistant strains have promoted the performance of antimicrobial interaction assays, using different combinations including vancomycin. Some studies have demonstrated synergistic effect for the combination of vancomycin and beta-lactams [19-21], and there is some evidence supporting its use in combination with aminoglycosides, in endocarditis [22,23].

In our study, synergy was evident for dicloxacillin or cephalothin in combination with amikacin, unfortunately, not in all cases the MICs in combination will be achieved therapeutically. In contrast with the results by Rochon-Edouard et al., we did not find a synergistic effect with the imipenem/vancomycin combination. The FIC indexes were inversely correlated with the MICs of imipenem (32 and 64 mg/L). The strains included in the present study required very high imipenem initial MICs (512 mg/L), and lower vancomycin concentrations (1–2 mg/L), therefore, results are poorly comparable. Results were similar for the vancomycin plus aminoglycoside combination (indifferent effect). One of the main obstacles to generalize the concept of the usefulness of antimicrobial combinations is the diversity of combinations in published studies [24-26]. In developing countries, the availability of new drugs, active against resistant strains is limited due to its cost, combinations of traditional antimicrobial agents that exhibit synergy or even additive activity could be an option.

## Conclusion

The best synergistic combination was cephalothin or dicloxacillin plus amikacin. The vancomycin combination with cephalothin or imipenem showed additivity. Vancomycin and amikacin had and indifferent effect. *In vivo *synergy and clinical efficacy cannot be predicted, but information of in *vitro *assays with resistant strains, could be useful to propose clinical studies to validate this information, most of all, in developing countries with a limited formulary.

## Competing interests

The author(s) declare that they have no competing interests.

## Authors' contributions

MVP drafted the manuscript, collected strain information and carried out identification of isolates. BLM carried out the antimicrobial combinations tests. FSS participated in the design and helped to draft the manuscript. GMN conceived of the study, participated in the coordination and helped to draft the manuscript. All authors read and approved the final manuscript.

## References

[B1] Christensen GD, Bisno AL, Parisi JT, McLaughlin B, Hester MG, Luther RW (1982). Nosocomial septicemia due to multiply antibiotic resistant *Staphylococcus epidermidis*. Ann Intern Med.

[B2] Martin MA, Pfaller MA, Wenzel RP (1989). Coagulase-negative staphylococcal bacteremia. Mortality and hospital stay. Ann Intern Med.

[B3] Panlilio AL, Culver DH, Gaynes RP, Banerjee S, Henderson TS, Tolson JS, Martone WJ (1992). Methicillin-resistant *Staphylococcus aureus *in US hospitals 1975–1991. Infect Control Hosp Epidemiol.

[B4] Suh K, Toye B, Jessamine P, Chan F, Ramotar K (1998). Epidemiology of methicillin-resistant *Staphylococcus aureus *in three Canadian tertiary-care centers. Infect Control Hosp Epidemiol.

[B5] Aires de Sousa M, de Lencastre H, Santos Sanches I, Kikuchi K, Totsuka K, Tomasz A (2000). Similarity of antibiotic resistant patterns and molecular typing properties of methicillin-resistant *Staphylococcus aureus *isolates widely spread in hospital in New York City and in a hospital in Tokyo, Japan. Micro Drug Resist.

[B6] Alpuche-Aranda C, Avila-Figueroa C, Espinoza-De los Monteros L, Gomez-Barreto D, Santos-Preciado JI (1989). Patrón de sensibilidad antimicrobiana de *Staphylococcus aureus *en un Hospital Pediátrico: prevalencia de resistencia a meticilina. Bol Med Hosp Infant Mex.

[B7] Giraud MC, Calva JJ, Huazano F, Ponce de Leon S, Ruiz-Palacios G (1986). Patrones de susceptibilidad a 19 antimicrobianos de gérmenes aislados de hemocultivos en un hospital de referencia de la ciudad de México. Rev Invest Clin.

[B8] Solórzano-Santos F, Miranda-Novales MG, Díaz-Ponce H, Leaños-Miranda B, Fajardo-Gutiérrez A (1994). Factores de riesgo para sepsis en pacientes pediátricos con infección por *Staphylococcus *coagulasa negativa. Bol Med Hosp Infant Mex.

[B9] Urdez-Hernandez E, Sifuentes-Osornio J, Calva JJ, Villalobos-Zapata Y (1999). Epidemiological and biological characteristics of methicillin-resistant staphylococcal infections in a Mexican Hospital. Arch Med Res.

[B10] Santos Sanches I, Mato R, de Lencastre H, Tomasz A, CEM/NET Collaborators and the International Collaborators. (2000). Patterns of multidrug resistance among methicillin-resistant hospital isolates of coagulase-positive and coagulase-negative Staphylococci collected in the International Multicenter Study Resist in 1997 and 1998. Microb Drug Res.

[B11] Moise-Broder PA, Sakoulas G, Eliopoulos GM, Schentag JJ, Forrest A, Moellering RC (2004). Accesory gene regulator group II polymorphism in methicillin-resistant *Staphylococcus aureus *is predictive of failure of vancomycin therapy. C I D.

[B12] CLSI (Clinical and Laboratory Standards Institute) (2005). Performance Standards for Antimicrobial Susceptibility testing. Fifteenth Informational Supplement CLSI document M100-S15 Pennsylvania USA.

[B13] Tenover FC, Jones RN, Swenson JM, Zimmer B, McAllister S, Jorgensen JH (1999). Methods for improved detection of oxacillin resistance in coagulase-negative staphylococci: results of a multicenter study. J Clin Microb.

[B14] Kohner P, Uhl J, Kolbert C, Persing D, Cockerill F (1999). Comparison of susceptibility testing methods with *mec A *gene analysis for determining oxacillin (methicillin) resistance in clinical isolates of *Staphylococcus aureus *and coagulase-negative *Staphylococcus *spp. J Clin Microbiol.

[B15] Velázquez-Meza ME, Aires de Sousa M, Echaniz-Aviles G, Solorzano-Santos F, Miranda-Novales G, Silva-Sanchez J, de Lencastre H (2004). Surveillance of methicillin-resistant *Staphylococcus aureus *in a pediatric hospital in Mexico City during a 7-year period (1997–2003):Clonal evolution and impact of infection control. J Clin Microbiol.

[B16] Norden CW, Wentzel H, Keleti E (1979). Comparison of techniques for measurement of in vitro antibiotic synergism. J Infect Dis.

[B17] Rochon-Edouard S, Pestel-Caron M, Lemeland JF, Caron F (2000). In vitro synergistic effects of double and triple combinations of b-lactams, vancomycin and netilmicin against methicillin-resistant *Staphylococcus aureus *strains. Antimicrob Agents Chemother.

[B18] Eliopolus GM, Moellering RC (2000). Antimicrobial combinations. In Antibiotics in Laboratory medicine.

[B19] Dawis MA, Isenberg HD, France KA, Jenkins SG (2003). *In vitro *activity of gatifloxacin alone and in combination with cefepime, meropenem, piperacillin and gentamicin against multidrug-resistant organisms. J Antimicrob Chemother.

[B20] Climo MW, Patron RL, Archer GL (1999). Combinations of vancomycin and b-lactams are synergistic against staphylococci with reduced susceptibilities to vancomycin. Antimicrob Agents Chemother.

[B21] Raymond J, Vedel G, Bergeret G (1996). In-vitro bactericidal activity of cefpirome in combination with vancomycin against *Staphylococcus aureus *and coagulase-negative staphylococci. J Antimicrob Chemother.

[B22] Le T, Bayer AS (2003). Combination antibiotic therapy for infective endocarditis. C I D.

[B23] Lee DG, Chun HS, Yim DS, Choi SM, Choi JH, Yoo JH, Shin WS, Kang MW (2003). Efficacies of vancomycin, arbekacin, and gentamicin alone or in combination against methicillin-resistant *Staphylococcus aureus *in an in vitro infective endocarditis model. Antimicrob Agents Chemother.

[B24] Snydman DR, McDermott LA, Jacobus NV (2005). Evaluation of in vitro interaction of daptomycin with gentamicin or beta-lactam antibiotics against *Staphylococcus aureus *and Enterococci by FIC index and timed-kill curves. J Chemother.

[B25] Lozniewski A, Lion C, Mory F, Weber M (2001). In vitro synergy between cefepime and vancomycin against methicillin-susceptible and – resistant *Staphylococcus aureus *and *Staphylococcus epidermidis*. J Antimicrob Chemother.

[B26] Raymond J, Vedel G, Bergeret M (1996). In-vitro bactericidal activity of cefpirome in combination with vancomycin against *Staphylococcus aureus *and coagulase-negative staphylococci. J Antimicrob Chemother.

